# Supplementary material for: Cloud BioLinux: pre-configured and on-demand bioinformatics computing for the genomics community
Source: BMC Bioinformatics. 2012 Mar 19;13:42. doi: 10.1186/1471-2105-13-42 (PMC3372431; doi:10.1186/1471-2105-13-42)
Supplement: Additional file 1 — Supplementary 1 Cloud BioLinux software documentation in the form of a mini, self-contained website. Users need to download and uncompress the .zip file, and open through a web browser the "index.html" file available on the main directory. (ZIP 1823 kb). [file 1471-2105-13-42-S1.ZIP › Cloud-BioLinux-Package-Documentation/docs/dolmove.html]

Bio-Linux Software Documentation Pages

Back to search form

## dolmove

|  |  |
| --- | --- |
| Name | dolmove |
| Description | **dolmove** is part of the PHYLIP package.  Copyright 1986-2004 by the University of Washington. Written by Joseph Felsenstein. Permission is granted to copy this document provided that no fee is charged for it and that this copyright notice is not removed.  **dolmove** is an interactive parsimony program which uses the Dollo and Polymorphism parsimony criteria. It is inspired on Wayne Maddison and David Maddison's marvellous program MacClade, which is written for Apple MacIntosh computers. DOLMOVE reads in a data set which is prepared in almost the same format as one for the Dollo and polymorhism parsimony program DOLLOP. It allows the user to choose an initial tree, and displays this tree on the screen. The user can look at different characters and the way their states are distributed on that tree, given the most parsimonious reconstruction of state changes for that particular tree.  The user then can specify how the tree is to be rearraranged, rerooted or written out to a file. By looking at different rearrangements of the tree the user can manually search for the most parsimonious tree, and can get a feel for how different characters are affected by changes in the tree topology.  **References:**  Felsenstein, J. 1993. PHYLIP (Phylogeny Inference Package) version 3.5c. Distributed by the author. Department of Genetics, University of Washington, Seattle.    Felsenstein, J. 1989. PHYLIP -- Phylogeny Inference Package (Version 3.2). Cladistics 5: 164-166. |
| Homepage | http://evolution.genetics.washington.edu/phylip.html |
| Remote Documentation | http://evolution.genetics.washington.edu/phylip/doc/dolmove.html |
